# Lower workforce participation is associated with more severe persisting breathlessness

**DOI:** 10.1186/s12890-022-01861-y

**Published:** 2022-03-18

**Authors:** Joseph Clark, Sungwon Chang, Irina Kinchin, Diana Ferreira, Slavica Kochovska, Deidre Morgan, Leanne M. Poulos, Miriam J. Johnson, Magnus Ekström, David C. Currow

**Affiliations:** 1 Wolfson Palliative Care Research Centre, Hull York Medical School, University of Hull, Hull, UK; 2 IMPACCT, Faculty of Health, University of Technology Sydney, Ultimo, NSW 2007 Australia; 3 Trinity College Dublin, the University of Dublin, Dublin, D02 PN40 Ireland; 4 College of Medicine and Public Health, Flinders University, Adelaide, SA 5042 Australia; 5 Research Centre for Palliative Care, Death and Dying (RePaDD), Flinders University, Adelaide, SA 5042 Australia; 6 Australian Centre for Airways Disease Monitoring (ACAM), Woolcock Institute of Medical Research, The University of Sydney, Sydney, NSW Australia; 7 Division of Respiratory Medicine and Allergology, Department of Clinical Sciences, Lund University, 22100 Lund, Sweden

**Keywords:** Persisting breathlessness, Cross-sectional population survey, Workforce participation, Income foregone

## Abstract

**Background:**

Not being able to work has negative health, social and financial consequences. Persisting breathlessness is prevalent in working-aged people. Is it associated with lower workforce participation? This study, using the South Australian Health Omnibus, aimed to explore associations between paid workforce participation and persisting breathlessness intensity, and economic impacts on income in people of working age.

**Methods:**

This cross-sectional study conducted face-to-face interviews with a random sample of adults in South Australia (n = 8916). Questions included key demographic data, workforce participation and the presence and intensity of persisting breathlessness. Data from working-aged respondents (20–65 years of age) were standardised to the census for regression analyses. Work was coded to paid full- or part-time work or ‘other’. Persisting breathlessness (more than three of the last six months) used the modified Medical Research Council breathlessness scale (aggregated to 0, 1, 2–4). Opportunity cost valuations compared annual income foregone by persisting breathlessness severity.

**Results:**

Of people interviewed, 6,608 were working-aged (49.9% male; 67.5% had post-secondary qualifications; 70.9% were in paid full- or part-time work; and 1.7% had mMRC score 2–4). Workforce participation dropped in working aged people with increasing breathlessness: mMRC 0, 70.6%; mMRC 1, 51.7%; mMRC 2–4, 20.3%. In the regression model, people with the most severe breathlessness were much less likely to work (OR 0.14; 95% CI 0.09, 0.22). Annual income foregone by people with persisting breathlessness was AU$10.7 billion (AU$9.1b for full-time and AU$1.6b for part-time work; range AU$5.9b, AU$49.7b).

**Conclusion:**

Worsening persisting breathlessness is associated with lower workforce participation with direct financial consequences, greatest for older males.

**Supplementary Information:**

The online version contains supplementary material available at 10.1186/s12890-022-01861-y.

## Background

Workforce participation depends on some relatively simple factors (age, sex, caring responsibilities) [1, 2] to more complex ones (social connectedness; finances; health status; the stimulation, satisfaction or challenge of the role; and the work environment). The latter includes the attitude of management to, and the willingness of workers to identify if they need to change roles because of changing physical abilities [3–5]. These factors are brought into sharper focus as people approach considering retirement [3, 4].

Meaningful workforce participation may contribute to a person’s health and wellbeing, including mental health [6–8] by impacting on people’s personal and social identities. People unable to work due to poor health often experience negative social and financial consequences [5, 9, 10] reflected in changing family roles, inability to maintain usual social functioning and lower incomes [11]. Optimising wellbeing by addressing avoidable causes of poor health is a fundamental aim of health systems which, in turn, may improve people’s workforce participation.

Persisting breathlessness is prevalent in the general population but commonly goes unrecognised [12, 13]. More than 2% of people in high-income countries experience persisting breathlessness, despite optimal management of the underlying cause(s) *and resulting in disability*.[14] Persisting breathlessness is frequently not volunteered by patients because they modify their lifestyle to minimise exertion [15]. It is often not identified by clinicians and, when identified, often goes unaddressed [16, 17]. This is despite the evidence-based non-pharmacological and pharmacological interventions which may reduce symptomatic persisting breathlessness and, potentially, improve function [18]. Persisting breathlessness is associated with: limits in daily activities; poorer quality of life; depression, anxiety; social isolation; reduced sexual activity; poorer sleep; and unplanned health service utilisation in primary care and emergency departments [13, 15, 19, 20]. Persisting breathlessness is predominantly attributed to chronic lung disease [21, 22], and is often present for long periods of time [21]. Given that persisting breathlessness impacts every aspect of personhood, it is important to understand its impact on working-aged people.

Most studies on workforce participation focus on demographic factors (age, sex) or diseases (cancer, motor neurone disease, para- or quadriplegia) [23, 24]. The relationship between the intensity of persisting breathlessness and workforce participation across the lifespan has not been explored at a whole-of-population level. In people in their 50 s, airway obstruction and breathlessness are independently associated with premature workforce cessation [25] and job instability [26]. To date, no studies to our knowledge have explored the relationship of persisting breathlessness with workforce participation in the population at large, irrespective of health service contact or specific diagnostic groups. If persisting breathlessness is associated with lower workforce participation, it would be even more important for clinicians to identify its presence, and to explore whether reducing its severity could help people re-engage in the workforce.

Population studies, independent of health service contact, are important for understanding disease and symptom burden. The aim of this study was to evaluate associations between self-reported workforce participation in working-aged people (20–65 years) and self-reported persisting breathlessness in the general population, and to assess any economic impact on people’s income.

## Methods

### Survey design

This population-based study used the South Australian Health Omnibus Survey (HOS) [14, 27], which was an annual, cross-sectional, multi-stage, clustered area, systematic sampling instrument. The HOS is a research platform where researchers pay to enter their questions annually with a maximum of approximately 200 questions each year. All researchers who subscribe in any given year are provided with standardised demographic data and census weightings that can be applied in subsequent analyses.

Data were collected by trained interviewers in structured, face-to-face interviews in respondents’ dwellings, lasting 60–90 min [27]. Identifying participants did not rely on health service contact. This study joined data on workforce participation and persisting breathlessness when both were asked (years 2006, 2015, 2017).

### Setting and respondents

South Australia has 6.9% (1.73 m) of the Australian population [28]. The survey was conducted in Spring annually, after piloting with 50 community members. Sampling identified a representative cohort from the metropolitan area and towns > 1000 population.

The sampling process involved two randomisations: randomly selected census collector districts (CCD); and randomly selected starting points within each CCD. From the randomly selected starting point, every tenth property was visited meaning not every address visited was a house (businesses, vacant land, hospitals, caravan parks and aged care facilities). Respondents were only drawn from houses. Interviewers would return up to five times if contact was not made initially. Participation rates were calculated on the number of people successfully contacted. One interview was conducted per household of the person ≥ 15 years of age who most recently had a birthday. If that person declined, the household was a ‘non-responder’.

### Data quality

Data were double entered, with follow-up of missing responses by telephone. Additionally, 10% of each interviewer’s respondents were re-contacted to confirm eligibility and ensure consistency of responses by re-answering some questions. Data were anonymised before release to researchers. No data were imputed.

Data were weighted to the Estimated Residential Population for South Australia by 5-year age groups, sex, rurality and household size derived from the Australian 2016 Census for regression analyses [29]. Data were then limited to respondents 20–65 years of age inclusive to reflect most of workforce as 65 was the age people could apply for the aged care pension at the time.

### Workforce participation

Respondents were asked to self-report select: full- and part-time (paid) work (employed by others or self-employed) with the balance of responses coded to ‘other’ (home duties; unemployed; retired; student; other; not working because of work related injury; or not working because of disability).

### Persisting breathlessness

**Fig. 1:**
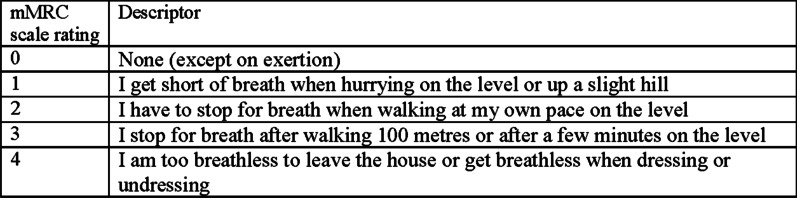
Modified Medical Research Council breathlessness scale

Persisting breathlessness was self-reported using the ordinal, five-level, modified Medical Research Council (mMRC) scale [30], combined with a descriptor of chronicity (lasting more than three of the last 6 months) [14, 19, 20]. The mMRC is a reliable, valid tool for population-based studies, categorising the extent of physical exertion before breathlessness limits ability using standard wording (Fig. 1) [30, 31]. Respondents were grouped (mMRC 0, mMRC 1 and, given small numbers, mMRC 2–4).

### Other covariates

Socio-demographic data were collected on age (stratified to 20–44 vs 45–65) [32], sex, place of residence (metropolitan/rural), country of birth (English speaking/other), domestic situation (married, de facto/other) and highest level of education (vocationally trained, university educated/other) and household income (≤/> AU$60k per annum) Respondents were asked their postcode to estimate their socio-economic status by quintile (Socio-Economic Index for Areas (SEIFA)).

### Statistical analysis

Analyses were conducted using SPSS for Windows Version 27.0 (SPSS Chicago, Il. USA, 2021). Respondents’ demographic characteristics were compared by workforce participation (full- or part-time versus other; full-time versus other) by Pearson’s chi-square for categorical variables. Workforce participation was analysed using logistic regression adjusting for socio-demographic factors. To identify potential confounders, all the covariates were examined in bivariate analyses with employment status [33, 34]. All significant covariates (p < 0.2) were included in a multiple logistic model. Stepwise deletion generated the adjusted model by individually dropping covariates with the highest p-value until all covariates had a p < 0.05. Active consideration was given to avoiding multi-collinearity to minimise bias from overfitting. Rurality was dropped from the final model, as were socio-economic status and household income given that the latter two were outcomes derived, in part, from income from paid employment. Associations with workforce participation were expressed as adjusted odds ratios (ORs) with 95% confidence intervals (CIs). The discriminatory power to distinguish between those who are and are not participating in the workforce was performed using a receiver operating characteristic (ROC) curve and area under the curve (AUC). A sensitivity analysis excluding 2006 survey data was performed to evaluate the robustness of the findings, given that there is at least a nine-year gap between 2006 and other surveys. All results use weighted data.

An opportunity cost valuation compared the difference in incomes of people with mMRC 0, 1 and mMRC 2–4 using current Australian Bureau of Statistics data [29] for average weekly incomes for full- and part-time work.

This was complemented with sensitivity analyses varying the: population levels of persisting breathlessness (two population estimates) [13]; workforce participation (± 10%); and average annual incomes (minimum wage rather than average).

The study’s reporting accords with STROBE guidelines (observational studies) [35].

## Results

The overall participation rate was 66.9% (Additional file 1: Fig. S1). Of the 8,955 people who were interviewed, weighted 6,608 (weighted to the national census) were aged 20–65 and included in analyses. Of those, 3,299 (49.9%) were male and 1656 (25.1%) lived in the state’s rural regions (Table 1). Two thirds of respondents had post-secondary qualifications. Women were more likely to report persisting breathlessness as were people in the 45–65 year old age group when compared to 20–44 year olds (p < 0.001 for both: Additional file 2: Table S1).

**Table 1:** Weighted sociodemographics and breathlessness by working status from a random sample of working aged (20–65), community-dwelling respondents in South Australia (n = 6,608)

	Working Status			p-value
	Total (n = 6,608)	Paid full- or part-time (n = 4688; 70.9%)	Other* (n = 1920; 29.1%)	
Age				
Mean (standard deviation)	41.9 (13.0)	41.1 (12.0)	43.6 (15.1)	< 0.001
	n (%)			
Younger (20–44)	3712 (56.2)	2741 (58.5)	971 (50.6)	0.001
Older (45–65)	2896 (43.8)	1947 (41.5)	949 (49.4)	
Sex				
Male	3299 (49.9)	2549 (54.4)	750 (39.1)	< 0.001
Female	3309 (50.1)	2139 (45.6)	1170 (60.9)	
egion of residence				
Non-metropolitan (rural, regional)	1656 (25.1)	1142 (24.4)	514 (26.8)	0.04
Metropolitan	4952 (74.9)	3546 (75.6)	1406 (73.2)	
Domestic situation				
Married/de facto	4443 (67.3)	3352 (71.5)	1091 (56.9)	< 0.001
Separated/divorced/widowed/never married	2163 (32.7)	1336 (28.5)	827 (43.1)	
Born in an English speaking country				
Yes	4876 (73.8)	3528 (75.3)	1348 (70.2)	< 0.001
No	1732 (26.2)	1160 (24.7)	572 (29.8)	
Vocationally trained/University educated				
Yes	4460 (67.5)	3485 (74.4)	975 (50.8)	< 0.001
No	2144 (32.5)	1201 (25.6)	943 (49.2)	
SEIFA (by quintile)				
1 (most disadvantaged)	1635 (24.8)	1028 (22.0)	607 (31.6)	< 0.001
2	1462 (22.1)	1025 (21.9)	437 (22.8)	
3	1213 (18.4)	885 (18.9)	328 (17.1)	
4	1150 (17.4)	889 (19.0)	261 (13.6)	
5 (most advantaged)	1141 (17.3)	855 (18.3)	286 (14.9)	
Annual household income				
> AU$60,000	2018 (37.7)	1087 (27.4)	931 (67.5)	< 0.001
≤ AU$60,000	3334 (62.3)	2886 (72.6)	448 (32.5)	
Breathlessness—mMRC				
0	6118 (92.6)	4453 (95.0)	1665 (86.7)	< 0.001
1	377 (5.7)	209 (4.5)	168 (8.8)	
2–4	113 (1.7)	26 (0.6)	87 (4.5)	

*‘Others’ includes home duties; unemployed; retired; student; other; not working because of work related injury; or not working because of disability; SEIFA—Social Economic Indexes for Area; mMRC—modified Medical Research Council breathlessness scale; not all sub-sections will add up to the column totals due to missing data

**Fig. 2:**
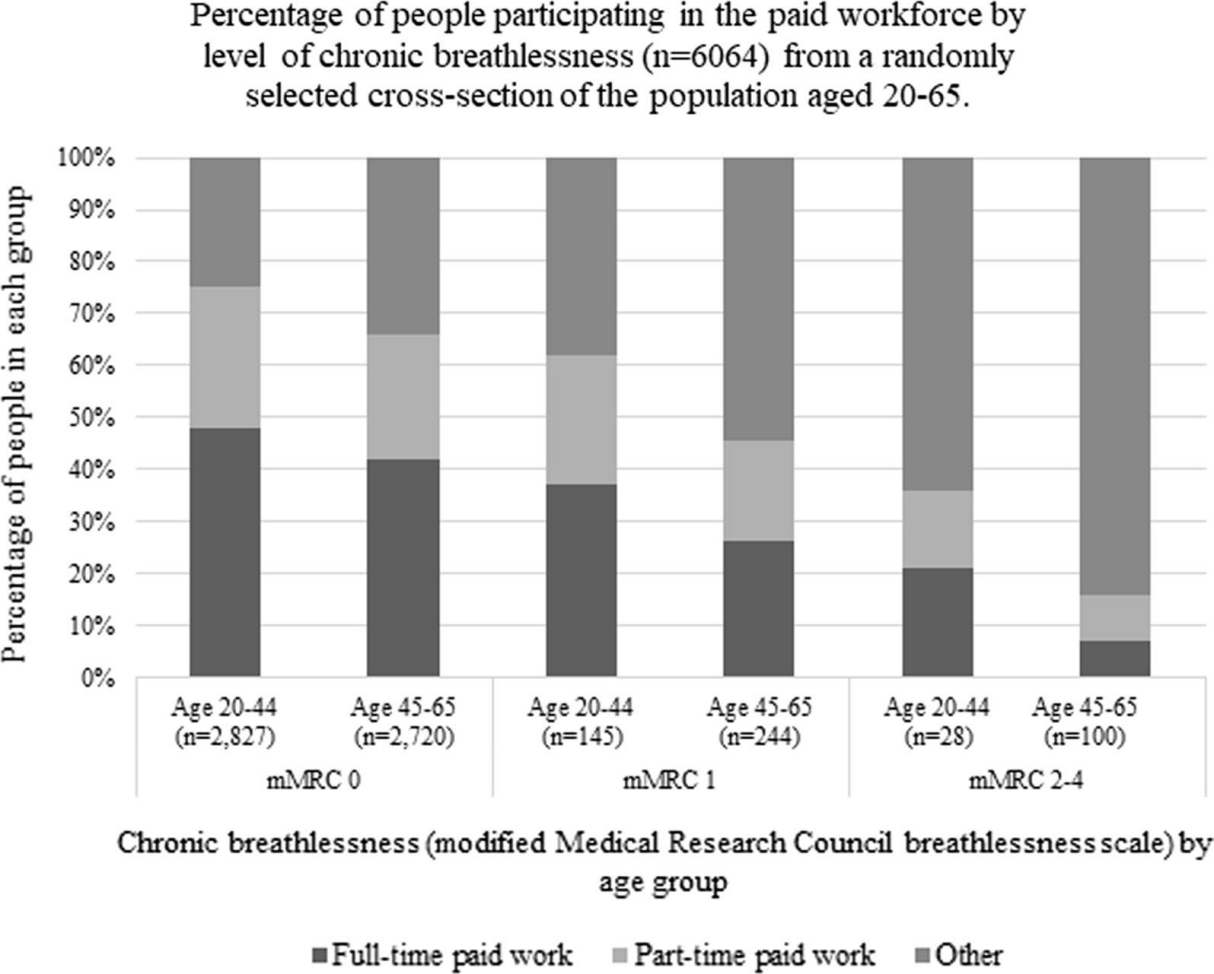
Percentage of people participating in the paid workforce by level of chronic breathlessness (n = 6064) from a randomly selected cross-section of the population aged 20–65

People who were in paid full- or part-time work (2741 (58.5%)) were younger (p < 0.001), more likely to be male (p < 0.001); while 113 (1.7%) respondents had mMRC 2–4 scores (Table 1). People with this level of breathlessness were far less likely to be in full- or part-time employment (4.5% versus 8.8% (mMRC 1); 0.6% vs 4.5% (mMRC 2–4); p < 0.001 for both comparisons). When broken down to ages 20–44 and 45–65, the disparities were even greater in the older age group (Fig. 2). Similar gradients were seen when people in full-time work were compared with all others. (Additional file 3: Table S2).

**Table 2:** Odds ratio of factors associated with work status (full- or part-time work compared with ‘other’; and full-time work versus ‘other’) from a random sample of working aged, community-dwelling respondents in South Australia (weighted data)

	Paid work (full- or part-time)	
	Model 1*	Model 2^#^
mMRC		
0	Ref	Ref
1	0.47 (0.38, 0.57)	0.54 (0.43, 0.67)
2–4	0.11 (0.07, 0.18)	0.14 (0.09, 0.22)
Survey		
2006	Ref	Ref
2015	0.93 (0.81, 1.06)	0.85 (0.74, 0.97)
2017	0.85 (0.74, 0.97)	0.81 (0.71, 0.93)
Mature age		0.71(0.63, 0.80)
Male		1.83 (1.63, 2.05)
Born in an English speaking country		1.88 (1.62, 2.19)
Vocationally/University trained		2.69 (2.39, 3.02)
Married / de facto		1.97 (1.75, 2.22)
Area under the (receiver operator) curve (AUC)	0.55 (0.54, 0.57)	0.70 (0.68, 0.7)

mMRC – modified Medical Research Council breathlessness scale

*Model 1: adjusted for the survey year only

^#^Model 2: adjusted for survey year, mature age, sex, Born in an English speaking country, vocationally/University trained, and in married/de facto

In the logistic regression models, people with the most severe breathlessness were far less likely to be in full- or part-time work (odds ratio (OR) 0.14; 95% confidence interval (CI) 0.09, 0.22) having adjusted for age, sex, country of birth, highest level of education, and domestic situation (Table 2). A similar difference was seen for the likelihood of being in full-time employment (OR 0.22; 95%CI 0.13, 0.39). These models had area under the receiver operator curves of 0.70 and 0.73 respectively (Table 2). Disparities in full- or part-time workforce participation worsened in the cohort with the most severe persisting breathlessness, among those aged 45–65 (OR 0.10; 95%CI 0.06, 0.19; Additional file 3: Table S3) and among males (OR 0.05; 95%CI 0.02, 0.13; Additional file 3: Table S4). Sensitivity analysis using 2015 and 2017 survey data demonstrated consistency in the direction and magnitude of findings. (Data not shown).

**Table 3:** The opportunity cost of clinically important breathlessness: workforce participation, chronic breathlessness and average annual income from a random sample of working aged, community-dwelling respondents in South Australia (n = 6064; unweighted data)

	Full-time	Part-time	All others	Total	Reference
mMRC 0,1 [A], n (%)	2628 (44.3%)	1491 (25.1%)	1817 (30.6%)	5936	Current sample
mMRC 2–4 [B], n (%)	13 (10.2%)	13 (10.2%)	102 (79.7%)	128	Current sample
Total	2641	1504	3423	6064	Current sample
Difference in workforce participation [C = A–B], %	34.1	14.9	Not valued		Estimated
Australian working age population [E], n				16,638,606	2020 average [33]
Prevalence of mMRC 2–4 among Australian working age population [D], %				2.1	Current sample
Australian working age population with mMRC 2–4 [F = D*E], n				349,411	Estimated
Average yearly income [G], AU$	76,076	30,004	Not valued		National data [34]
Income forgone (and assuming no other income) [CxFxG], AU$ billion	9.1	1.6	Not valued	10.6	Estimated

**Table 4:** Sensitivity analysis of the opportunity cost of clinically important breathlessness: workforce participation, chronic breathlessness and average annual income from a random sample of working aged, community-dwelling respondents in South Australia (n = 6064; unweighted data) varied by prevalence of chronic breathlessness, proportion of these people in full- or part-time work and by income

	Full-time (AU$ bn)	Part-time (AU$ bn)	Total (AU$ bn)	References
Prevalence of mMRC 2–4 among Australian working age population				
Baseline (2.1%)	9.1	1.6	10.7	Current sample
Sensitivity 1 (9.8%)	42.4	7.3	49.7	Poulos et al
Proportion of mMRC 2–4 in full and part-time workforce				
Sensitivity 3 (Increase by 10% (11.2%))	8.8	1.5	10.3	Expert advice
Baseline (10.2%)	9.1	1.6	10.7	Current sample
Sensitivity 4 (Decrease by 10% (9.2%))	9.3	1.7	11.0	Expert advice
Average weekly income				
Sensitivity 5 (National minimum wage AU$772.60/38 h week)	4.8	1.1	5.9	Fairwork Australia [35]
Baseline (AU$1,463/week for full-time and AU$577/week for part-time)	9.1	1.6	10.7	Australian Bureau of Statistics [34]

Economically, estimates of opportunity cost valuations at a population level reflected reduced full-time workforce participation in people with the most severe persisting breathlessness (mMRC 2–4; 10.2% in full-time work) compared to mMRC 0,1 (44.3% in full-time work; a deficit of 34.1%; 184,945 people; Table 3) and for part-time work, this was a difference of 14.9% (25.1–10.2%; 80,812 people). Australia’s 20–65 year old population in 2020 was 16,638,606 [36]. Average full-time annual income was AU$76,076, and average part-time income AU$30,004 [37]. Annual income foregone by people with persisting breathlessness for full-time work was AU$9.1billion and for part-time work, AU$1.6b totalling AU$10.7b.

Sensitivity analyses varying all key parameters resulted in the total opportunity costs of more severe breathlessness ranging from AU$5.9billion to AU$49.7billion (Table 4). The largest variance in estimates related to the uncertainty about the proportion of mMRC 2–4 among the Australian working-age population.

## Discussion

The main finding is that worsening persisting breathlessness is associated with lower paid workforce participation among people of working age in the community. This adds to the significant costs related to the syndrome already borne by individuals, families and societies. Older workers (aged 45–65) were more likely to have persisting breathlessness and less likely to participate in the workforce. The estimated earnings foregone for paid employment due to severe persisting breathlessness were AU$10.6b (range AU$5.9b to AU$49.7b). The major driver for the wide range was driven almost entirely by the proportion of the population with more severe persisting breathlessness. This defines the extent and impact of limited workforce participation in people with persisting breathlessness, building on earlier work suggesting such a relationship [14].

Persisting breathlessness is most often attributed to respiratory disease [21], most of which will be caused by chronic obstructive pulmonary disease (COPD). In 2018, COPD generated the fourth greatest disease burden in Australia, accounting for 3.5% of the total [38]. People with COPD and other causes of persisting breathlessness often experience this disabling syndrome for years or decades [21]. Therefore, it is likely that workforce participation will be affected for a large portion of the population living with COPD.

Persisting breathlessness is not distributed evenly across socio-economic strata and is much more likely to affect people in lower strata, reflecting, in part, smoking rates (which, in Australia, are 3.7 times higher in the lowest socio-economic quintile compared to the highest) [39] and occupational exposures. It is likely, therefore, that the disadvantage from lower rates of participation in paid work as a result of persisting breathlessness is also felt disproportionately by people from the lower quintiles of socio-economic status, reflecting the sensitivity analysis of the opportunity cost model when using the minimum wage.

Persisting breathlessness can be reduced for many people using evidence-based non-pharmacological and pharmacological interventions. The provision of optimal symptom management may improve a person’s function and ability to participate in everyday activities and, by extension, potentially workforce participation.

### Strengths

This cross-sectional study is of interviews with randomly selected members of the general public and does not rely on clinicians’ referral nor identification through clinical services. It builds on a strong research platform of previous annual face-to-face surveys. The findings have strong face validity, with consistency in direction and magnitude of findings through sensitivity analyses.

### Limitations

This study cannot define a cause and effect relationship between persisting breathlessness and workforce participation. The results demonstrate a strong association between the two.

This paper cannot account for changes in work capability for people who have managed to stay in the workforce with persisting breathlessness, nor can it account for ‘presenteeism’ where people are attending work but whose productivity is reduced because of chronic problems [40]. This study does not account for other household members who have to forego paid work to care for people with persisting breathlessness [8] nor the value of unpaid workforce participation, such as volunteering. The other aspect of workforce participation that this study cannot define are people who want to participate in unpaid work for its mental and social benefits but are unable to because of their persisting breathlessness. The study cannot account for people working part-time who would have preferred to work more hours up to full-time. Such exclusions underestimate of the true impacts of severe breathlessness.

There may be residual confounding due to other socio-economic factors that were not collected as part of these surveys. Future work should include a wider range of socio-economic indices. Likewise, smoking data were not available to the researchers for all of the years. Previous work using a similar methodology did not find a strong relationship between dichotomous questions about smoking and persisting breathlessness [41].

These data cannot explore other reasons for people who happen to have persisting breathlessness disengaging from the paid workforce. In likelihood, many people will have a number a comorbidities that may contribute to lower workforce participation. Solely reversing persisting breathlessness therefore may not increase workforce participation.

Self-reported data regarding workforce participation might be subject to social desirability bias. Respondents were assured that their data would be anonymised before release to researchers to minimise this risk.

Although run annually, there is a nine-year gap between two of the surveys. Given that these are the first population-based data exploring this relationship, it was decided to use all three years of available data (given that still only 26 respondents of the 128 with mMRC 2–4 were in paid work). Weighting all data to the most recent census helps to limit impacts from changes in temporal patterns of persisting breathlessness or workforce participation.

### Implications for policy

As populations age, the proportion of older adults expected or required to remain employed will increase. Given the relationship of persisting breathlessness and age to workforce participation, its impact is likely to increase in the decades ahead.

The environment and flexibility of workplaces together with the support of the supervisors and colleagues impacts on people’s ability and willingness to participate in the workforce when they have chronic or progressive illnesses [10, 42]. Employers have responsibilities to their workforces to take reasonable steps to facilitate continuing workforce participation in the face of disabilities such as persisting breathlessness. As technology-enabled work options such as working remotely continue to evolve, the choices for people with chronic or progressive health conditions including persisting breathlessness should widen. This will allow a greater proportion of people being able to work from home or in work environments that are sympathetic to the health and wellbeing of employees [43].

### Implications for practice

Given the recent recognition of persisting breathlessness as a syndrome [12], work needs to be done with clinicians to improve its identification, assessment and symptomatic management. Persisting breathlessness most often has an insidious onset, which progressively reduces function over time. Many people reduce activities and exertion to cope, despite its increasing impacts on daily lives. That persisting breathlessness is associated with limiting economic activity amongst people of working age should elevate the importance of actively seeking a history from people who are more likely to experience persisting breathlessness. If not, persisting breathlessness will continue to be invisible to clinicians given that patients are unlikely to volunteer its presence or extent. This study suggests that clinicians should ask routinely about work history, work foregone and limitations that impair working abilities in order to align as closely as possible with each person’s occupational goals.

### Implications for further research

Given the massive social and economic consequences of persisting breathlessness associated with lower workforce participation, future work should focus on whether better symptom control (assuming optimal clinical management of underlying clinical conditions) can enable better day-to-day function and therefore potentially better engagement with the workforce for some people. This may include re-entry to work (paid or unpaid) or an increase in the number of hours worked. If such changes could be shown, this could have profound effects on people with persisting breathlessness, their families and the health systems that provide their care.

## Conclusions

Given the higher prevalence of persisting breathlessness in women, these findings may be one factor that systematically worsens the employment security and financial wellbeing of women. Persisting breathlessness may result in greater burden and uncertainty, especially as one’s working life draws to a close. Further studies are needed to elicit qualitative experiences of breathlessness in relation to workforce participation, to identify modifiable causes of working days lost in different settings. Additionally, the extent to which persisting breathlessness impacts on carers’ capacity to work needs to be investigated.

## Supplementary Information

**Additional file 1: Fig. S1.** Flow diagram for participation in the face-to-face interviews of the South Australian Health Omnibus where questions on workforce participation and chronic breathlessness were asked in the same years (2006, 2015, 2017).

**Additional file 2: Table S1.** Proportions of people by sex and, separately, age group with persisting breathlessness from random population sample collected in face-to-face interviews from the South Australian Health Omnibus. (n = 6064).

**Additional file 3: Tables S2.** Working status (full-time compared with ‘other’) by demographic variables in a random sample of community-dwelling, working-aged people (n = 6064) in South Australia (unweighted data). **Table S3.** Odds ratios of complete model of associations of breathlessness with working status stratified by age in a random sample of community-dwelling, working-aged people (n = 6064) in South Australia (weighted data). **Table S4.** Odds ratios of complete model of associations of breathlessness with working status stratified by sex in a random sample of community-dwelling, working-aged people (n = 6064) in South Australia (weighted data).

## Data Availability

The datasets used and/or analysed during the current study are available from the corresponding author on reasonable request.

## Abbreviations

CCD: Census collector districts
CI: Confidence intervals
COPD: Chronic obstructive pulmonary disease
HOS: Health Omnibus Survey
mMRC: Modified Medical Research Council
OR: Odds ratios
ROC: Receiver operator curves
SEIFA: Socio-Economic Index for Areas
