# Supplementary material for: Lower workforce participation is associated with more severe persisting breathlessness
Source: BMC Pulm Med. 2022 Mar 18;22:93. doi: 10.1186/s12890-022-01861-y (PMC8933889; doi:10.1186/s12890-022-01861-y)
Supplement: Supplementary file 1 — Additional file 1: Fig. S1. Flow diagram for participation in the face-to-face interviews of the South Australian Health Omnibus where questions on workforce participation and chronic breathlessness were asked in the same years (2006, 2015, 2017). [file 12890_2022_1861_MOESM1_ESM.docx]

Supplementary Figure 1

Flow diagram for participation in the face-to-face interviews of the South Australian Health Omnibus where questions on workforce participation and chronic breathlessness were asked in the same years (2006, 2015, 2017)

|  |  | Total |
| --- | --- | --- |
| **Initial sample drawn – physical addresses** | | **16,200** |
| **Sample loss** | Vacant houses, businesses, vacant land | 334 |
| **Remaining sample** |  | **15,866** |
| **Non-response** | Declined (not interested, too busy) | 3,918 |
|  | *Contact could not be established after 6 visits | 2,106 |
|  | Respondent unable to speak English | 232 |
|  | *Selected respondent away for the duration | 183 |
|  | Illness / mental incapacity | 268 |
|  | *Locked gate / unable to access building | 188 |
|  | *Ferocious dog | 13 |
|  | Terminated interview | 3 |
| **Contact could be made with the household** | | **13,376** |
| **Total interviews** | | **8,955** |
| **Participation percentage** | *excluded from denominator as no contact could be made | **66.9%** |
