# Supplementary material for: Lower workforce participation is associated with more severe persisting breathlessness
Source: BMC Pulm Med. 2022 Mar 18;22:93. doi: 10.1186/s12890-022-01861-y (PMC8933889; doi:10.1186/s12890-022-01861-y)
Supplement: Supplementary file 2 — Additional file 2: Table S1. Proportions of people by sex and, separately, age group with persisting breathlessness from random population sample collected in face-to-face interviews from the South Australian Health Omnibus. (n = 6064). [file 12890_2022_1861_MOESM2_ESM.docx]

Supplementary table 1.

Proportions of people by sex and, separately, age group with persisting breathlessness from random population sample collected in face-to-face interviews from the South Australian Health Omnibus. (n=6064)

|  |  | mMRC n, % | | |  |
| --- | --- | --- | --- | --- | --- |
|  |  | 0 | 1 | 2-4 | p value |
| Sex | Female | 3,204  89.9% | 270  7.6% | 89  2.5% | <0.001 |
|  | Male | 2,343  93.7% | 119  4.8% | 39  1.6% |  |
| Age group | 20-44 | 2,827  94.2% | 145  4.8% | 28  0.9% | <0.001 |
|  | 45-65 | 2,720  88.8% | 244  8.0% | 100  3.3% |  |

…
