# Supplementary material for: Lower workforce participation is associated with more severe persisting breathlessness
Source: BMC Pulm Med. 2022 Mar 18;22:93. doi: 10.1186/s12890-022-01861-y (PMC8933889; doi:10.1186/s12890-022-01861-y)
Supplement: Supplementary file 3 — Additional file 3: Tables S2. Working status (full-time compared with ‘other’) by demographic variables in a random sample of community-dwelling, working-aged people (n = 6064) in South Australia (unweighted data). Table S3. Odds ratios of complete model of associations of breathlessness with working status stratified by age in a random sample of community-dwelling, working-aged people (n = 6064) in South Australia (weighted data). Table S4. Odds ratios of complete model of associations of breathlessness with working status stratified by sex in a random sample of community-dwelling, working-aged people (n = 6064) in South Australia (weighted data). [file 12890_2022_1861_MOESM3_ESM.docx]

Supplementary Table 2

Working status (full-time compared with ‘other’) by demographic variables in a random sample of community-dwelling, working-aged people (n=6064) in South Australia (unweighted data).

|  | | Work status | | | p-value |
| --- | --- | --- | --- | --- | --- |
|  | | Total  (n=6064) | Paid full-time  (n=2641) | Other*  (n=3423) |  |
| Age | mean (standard deviation) | 44.3 (12.9) | 42.7 (11.7) | 45.6 (13.6) | <0.001 |
|  |  | n (%) | | |  |
|  | Older (45-65) | 3000 (49.5) | 1420 (53.8) | 1580 (46.2) | <0.001 |
|  | Younger (20-44) | 3064 (50.5) | 1221 (46.2) | 1843 (53.8) |  |
| Sex | | | | | |
| Male | | 2501 (41.2) | 1551 (58.7) | 950 (27.8) | <0.001 |
| Female | | 3563 (58.8) | 1090 (41.3) | 2473 (72.2) |  |
| Region of residence | | | | | |
| Non-metropolitan (rural, regional) | | 1480 (24.4) | 585 (22.2) | 895 (26.1) | <0.001 |
| Metropolitan | | 4584 (75.6) | 2056 (77.8) | 2528 (73.9) |  |
| Domestic situation | | | | | |
| Married / *de facto* | | 3766 (62.1) | 1717 (65) | 2049 (59.9) | <0.001 |
| Separated/divorced/widowed/never married | | 2295 (37.9) | 924 (35) | 1371 (40.1) |  |
| Born in an English speaking country | | | | | |
| Yes | | 5190 (85.6) | 2289 (86.7) | 2901 (84.8) | <0.001 |
| No | | 874 (14.4) | 352 (13.3) | 522 (15.2) |  |
| Vocationally trained / University educated | | | | | |
| Yes | | 4045 (66.7) | 2027 (76.8) | 2018 (59) | <0.001 |
| No | | 2015 (33.3) | 613 (23.2) | 1402 (41) |  |
| SEIFA (by quintile) | | | | | |
| 1 (most disadvantaged) | | 1556 (25.7) | 595 (38.25) | 961 (61.81) | <0.001 |
| 2 | | 1320 (21.8) | 553 (41.9) | 767 (58.1) |  |
| 3 | | 1088 (18) | 501 (46.0) | 587 (54.02) |  |
| 4 | | 1052 (17.4) | 495 (47.1) | 557 (52.9) |  |
| 5 (most advantaged) | | 1043 (17.2) | 495 (47.5) | 548 (52.5) |  |
| Annual household income | | | | | |
| >AU$60,000 | | 2789 (55) | 1742 (62.5) | 1047 (37.5) | <0.001 |
| ≤AU$60,000 | | 2279 (45) | 605 (26.5) | 1674 (73.5) |  |
| Breathlessness – mMRC | | | | | |
| 0 | | 5547 (91.5) | 2510 (45.2) | 3037 (54.8) | <0.001 |
| 1 | | 389 (6.4) | 118 (30.3) | 271 (69.7) |  |
| 2-4 | | 128 (2.1) | 13 (10.2) | 115 (89.8) |  |

* ‘others’ includes part-time paid work, home duties; unemployed; retired; student; other; not working because of work related injury; or not working because of disability; SEIFA - Social Economic Indexes for Area; mMRC - modified Medical Research Council breathlessness scale; not all sub-sections will add up to the column totals due to missing data

Supplementary Table 3

Odds ratios of complete model of associations of breathlessness with working status stratified by age in a random sample of community-dwelling, working-aged people (n=6064) in South Australia (weighted data).

Association of breathlessness with working status stratified by age weighted data

|  | Paid work (full- or part-time) | | Paid work (full-time only) | |
| --- | --- | --- | --- | --- |
|  | 20-44 years | 45-65 years | 20-44 years | 45-65 years |
| mMRC  0  1  2-4 | Ref  0.66 (0.47, 0.93)  0.26 (0.12, 0.58) | Ref  0.45 (0.33, 0.61)  0.1 (0.06, 0.19) | Ref   \| 0.87 (0.62, 1.22)  0.62 (0.26, 1.47) \|  \| \| --- \| --- \| \|  \|  \| | Ref  0.52 (0.37, 0.72)  0.12 (0.05, 0.28) |
| Survey  2006  2015  2017 | Ref   \| 0.78 (0.64, 0.95)  0.86 (0.70, 1.04) \| \| \| --- \| --- \| \|  \| \| | Ref  1.02 (0.83, 1.25)  0.8 (0.65, 0.98) | Ref   \| 0.8 (0.67, 0.95) \| \| --- \| \| 0.77 (0.64, 0.91) \| | Ref   \| 0.91 (0.75, 1.11) \| \| --- \| \| 0.81 (0.66, 0.99) \| |
| Male | 0.78 (0.64, 0.95) | 0.72 (0.61, 0.85) | 0.20 (0.17, 0.23) | 0.25 (0.21, 0.29) |
| Born in an English speaking country | 2.24 (1.85, 2.73) | 1.49 (1.17, 1.89) | 1.78 (1.48, 2.15) | 1.12 (0.88, 1.42) |
| Vocationally trained / University educated | 2.88 (2.44, 3.40) | 2.56 (2.16, 3.03) | 2.27 (1.93, 2.67) | 2.11 (1.78, 2.51) |
| Married / *de facto* | 2.38 (2.02, 2.80) | 1.6 (1.33, 1.93) | 2.11 (1.81, 2.46) | 1.32 (1.09, 1.59) |
| Area under the (receiver operator) curve (AUC) | 0.70 (0.68, 0.72) | 0.68 (0.66, 0.70) | 0.72 (0.70, 0.74) | 0.70 (0.68, 0.72) |

Supplementary Table 4

Odds ratios of complete model of associations of breathlessness with working status stratified by sex in a random sample of community-dwelling, working-aged people (n=6064) in South Australia (weighted data).

|  | Paid work (full- or part-time) | | Paid work (full-time only) | |
| --- | --- | --- | --- | --- |
|  | Male | Female | Male | Female |
| mMRC  0  1  2-4 | Ref  0.49 (0.34, 0.71)  0.05 (0.02, 0.13) | Ref  0.56 (0.43, 0.75)  0.21 (0.12, 0.35) | Ref   \| 0.56 (0.39, 0.8)  0.08 (0.03, 0.22) \|  \| \| --- \| --- \| \|  \|  \| | Ref  0.74 (0.54, 1.01)  0.44 (0.23, 0.82) |
| Survey  2006  2015  2017 | Ref   \| 1.02 (0.82, 1.27)  0.83 (0.67, 1.03) \| \| \| --- \| --- \| \|  \| \| | Ref  0.78 (0.65, 0.94)  0.8 (0.66, 0.96) | Ref   \| 0.89 (0.73, 1.07) \| \| --- \| \| 0.68 (0.57, 0.83) \| | Ref   \| 0.8 (0.67, 0.97) \| \| --- \| \| 0.89 (0.74, 1.07) \| |
| Older Age (45-65) | 0.46 (0.38, 0.56) | 0.92 (0.79, 1.07) | 0.58 (0.49, 0.69) | 0.90 (0.77, 1.05) |
| Born in an English speaking country | 1.88 (1.49, 2.36) | 1.96 (1.60, 2.39) | 2.01 (1.63, 2.47) | 1.15 (0.93, 1.42) |
| Vocationally trained / University educated | 2.41 (2.01, 2.88) | 2.98 (2.54, 3.48) | 2.13 (1.8, 2.51) | 2.29 (1.92, 2.72) |
| Married / *de facto* | 3.32 (2.75, 4.01) | 1.40 (1.19, 1.64) | 3.88 (3.29, 4.59) | 0.77 (0.65, 0.9) |
| Area under the (receiver operator) curve (AUC) | 0.72 (0.70, 0.74) | 0.67 (0.65, 0.69) | 0.70 (0.68, 0.72) | 0.62 (0.61, 0.65) |
